# Balancing care needs - a qualitative study on prehospital emergency nurses’ experiences of providing self-care advice and home referrals for frail older patients

**DOI:** 10.1186/s12873-025-01355-0

**Published:** 2025-09-22

**Authors:** Gabriella Norberg Boysen, Elin Svensson, Caroline Heldtander, Johan Herlitz, Agnes Olander

**Affiliations:** 1 Faculty of Health and Life Sciences, Linnaeus University, Växjö, Sweden; 2 PreHospen – Centre for Prehospital Research, University of Borås, Borås, Sweden; 3 Department of Prehospital Emergency Care, Sahlgrenska University Hospital, Gothenburg, Sweden; 4 Faculty of Caring Science, Work Life and Social Welfare, University of Borås, Borås, Sweden; 5 Faculty of Health and Society, Kristianstad University, Kristianstad, Sweden; 6 The Research Environment – Patient Reported Outcomes – Clinical Assessment Research and Education (PRO-CARE), Kristianstad University, Kristianstad, Sweden

**Keywords:** Frail older patient, Prehospital emergency care, Prehospital emergency nurse, Self-care advice, Interview, Qualitative

## Abstract

**Background:**

The aging population has led to a growing number of frail older patients in prehospital emergency care. These patients often present with complex healthcare needs, posing significant challenges for prehospital emergency nurses (PENs) when assessing the appropriateness of providing self-care advice and recommending that patients remain at home. This study, therefore, aimed to explore the experiences of PENs in giving self-care advice and referring frail older patients to remain at home when this approach is considered the most appropriate course of action.

**Methods:**

An exploratory qualitative design was employed, which was based on individual semistructured interviews with ten PENs from an ambulance service organization in southwestern Sweden. The data were analysed via inductive qualitative content analysis, following the approach described by Elo and Kyngäs.

**Results:**

The analysis resulted in one main category: *Professional discretion and coordinated support for sustainable decision making*, along with three generic categories. The findings highlight key dilemmas faced by PENs: navigating uncertainty in frail older patients’ cognitive and functional abilities, balancing patient autonomy and self-determination with safety concerns, and managing the ethical tension between respecting dignity and preventing harm. PENs also highlighted dilemmas in communication, where striving for clarity and trust was hampered by language barriers. They further experienced challenges in negotiating power and responsibility with frail older patients and relatives. Limited resources, unclear care structures, and insufficient collaboration with other healthcare providers created additional organizational dilemmas, constraining PENs’ ability to make confident, ethically sustainable decisions.

**Conclusion:**

This study underscores the complexity of decision-making for PENs when giving self-care advice and referring frail older patients to remain at home. Ensuring safe and sustainable care requires thorough assessments, clear communication, clinical expertise, empathy, and interprofessional collaboration, while future research should also address patients’ perspectives and the organizational factors influencing PENs’ decisions.

**Clinical trial registration:**

Not applicable.

**Supplementary Information:**

The online version contains supplementary material available at 10.1186/s12873-025-01355-0.

## Background

In 2022, Sweden had over 2.6 million residents aged 60 years or older, representing approximately one quarter of the national population and indicating a significant increase compared to five decades ago [1]. According to the World Health Organization, the number of people aged 60 years and older worldwide is projected to rise from 1 billion in 2019 to 1.4 billion by 2030 and further to 2.1 billion by 2050 [2]. This demographic shift has led to more frail older patients requiring prehospital emergency care; in Sweden, nearly 60% of those treated by ambulance services are frail older adults [3], and a recent European study reported that about 40% of emergency patients ≥ 65 years had at least mild frailty [4]. Frailty, understood as a multidimensional vulnerability across physical, psychological, and social domains, is often combined with multimorbidity and complex social circumstances, making decision-making particularly demanding for prehospital emergency nurses (PENs) [5]. While prehospital emergency care has traditionally focused on transporting patients to hospital emergency departments, the role of PENs has expanded to include assessment, treatment, and referral to alternative care levels, such as supporting patients to remain at home through tailored self-care advice [6]. This shift aims to reduce risks associated with emergency department visits, where frail older patients face long waits and complications such as delirium, falls, infections, and functional decline in overcrowded settings [7, 8]. However, another study indicated that redirecting patients to primary care may also involve safety risks, with some experiencing adverse events post-referral [9]. These findings underscore the importance of implementing alternative care pathways with careful consideration to avoid unintended harm [7, 9, 10]. This need is further emphasized by the fact that modern emergency departments are not well suited to meet the needs of frail older patients, as their operations tend to prioritize medical-technical care over patient-centered care for less critical conditions. Emergency departments employ a triage system where the most life-threatening cases are treated first to quickly free up resources. Consequently, frail older patients who do not meet the criteria for urgent treatment may experience prolonged and resource-intensive stays, often without adequate access to necessary care [8]. Therefore, in certain cases, it may be appropriate to consider allowing these patients to remain at home with adequate self-care advice, provided that this does not compromise their safety [11].

In summary, the increasing number of frail older patients in prehospital emergency care, combined with the evolving role of PENs in making referral decisions, highlights the challenges they face, particularly when they decide to refer these patients to remain at home with self-care advice. Understanding these experiences is crucial for addressing PENs’ difficulties in this decision-making process. Consequently, this study aimed to explore PENs’ experiences in giving self-care advice and deciding to refer frail older patients to remain at home when this approach is considered most appropriate.

## Methods

### Study design

The present study employed an exploratory, qualitative approach. Data were collected through individual semi-structured interviews, guided by an interview guide with open-ended, follow-up, and probing questions to encourage rich descriptions (Supplementary File 1). An inductive content analysis was conducted to capture and deepen the understanding of participants’ experiences related to the phenomenon [12]. In this study, the authors followed the COREQ guidelines and checklist (Supplementary File 2) [13].

### Study settings

One ambulance service organization in southwestern Sweden was included, covering a catchment area of approximately 930 km² with 690,000 inhabitants and handling around 80,000 missions annually. The participating healthcare professionals were PENs. In Sweden, PENs hold an independent responsibility for assessing patients, initiating care, and deciding whether transportation to the emergency department is necessary or whether the patient can remain at home with self-care advice.

This decision-making authority is regulated within the Swedish healthcare system, where ambulance services are part of the publicly funded regional healthcare organizations. PENs work in close collaboration with community healthcare teams, primary care, home care, home nursing services, and hospital-based care. Community healthcare teams provide multidisciplinary home visits and follow-up for patients with complex needs. Primary care refers to general medical services provided at health centers by physicians and nurses, often serving as the first point of contact in the Swedish healthcare system. Home care (municipal support for daily activities) and home nursing services (nursing-specific interventions such as medication or wound care) are distinct but often overlap.

### Participants

The study included ten PENs employed in the ambulance service organization, selected through a convenience sampling method [14]. PENs in Sweden are registered nurses with specialist training in prehospital emergency care (three years of nursing education followed by a one-year specialist program, leading to a Master of Science degree). The inclusion criterion was at least three months of experience as a PEN in prehospital emergency care. Exclusion criteria included any PEN with whom the authors had a personal relationship to avoid potential influence on interview dynamics and to ensure participant openness.

PENs were first provided with brief information about the study by the Head of Unit via their workplace email. Those interested in participating were asked to contact the author ES or CH directly via email or phone to receive more detailed information. At this stage, two individuals declined to participate due to discomfort with the interview process. In total, ten PENs agreed to take part, consisting of six men and four women, aged 27–57 years, with professional experience ranging from nine months to eleven years.

Written approval to conduct the interviews was obtained from the Head of Department responsible for the ambulance service organization. Verbal and written information about the study was provided, and all participants gave written informed consent prior to participation. They were assured of confidentiality and informed of their right to withdraw from the study at any time without providing a reason.

### Data collection

A pilot interview was conducted to test the semi-structured interview guide with open-ended questions [12]. Based on this pilot, the questions were refined to improve clarity and to reduce the risk of influencing participants’ responses. The pilot interview was not included in the final data set.

The main interviews were conducted from February to September 2024 by the authors ES and CH. All interviews took place in person at the participants’ workplaces. In total, ten interviews were conducted, lasting between 15 and 31 min each, with a total interview time of 229 min. During the interviews, both authors were present: one author led the interview while the other listened and asked follow-up questions when necessary. The interviews were digitally recorded and transcribed verbatim, including pauses, sounds, sighs, and emotional expressions, to capture the full range of participants’ experiences. Data collection continued until no new significant information or variation in the data emerged, and data saturation was considered to have been reached.

### Data analysis

The analysis was conducted via an inductive qualitative content analysis based on the method outlined by Elo and Kyngäs [12], following three steps: preparation, organization, and reporting. The preparation phase began with the transcription of the texts from all the interviews. The transcribed text was repeatedly read through to develop a comprehensive understanding of the content. In the organization phase, meaning units relevant to the study’s aim were identified, and their significance was noted in the margins of the text material. The meaning units were then transferred to a separate Word document for further organization. Within the meaning units, experiences and emotions related to the study’s aim were identified. All experiences and emotions in the text were color-coded and marked with a specific code. The text was then condensed, retaining the codes, which summarized the meaning of each sentence. These codes were subsequently sorted into subcategories. Subcategories were then grouped when they were found to have the same or similar meanings. The subcategories with the same or similar meanings were combined to form a group with a specific heading. The goal was to reduce the number of subcategories by merging them into broader, overarching generic categories. The abstraction process continued as long as it was feasible and reasonable, and ultimately, the generic categories were consolidated into a main category. The primary analysis was conducted by ES and CH, who carried out the initial coding and categorization of the interview data. This preliminary analysis was then discussed with GBN to refine the interpretation. To further deepen the analysis, GBN and AO re-examined the entire dataset. This reanalysis went beyond validation and led to the development of new and more nuanced categories. The results of this process were finally discussed among all authors until consensus was reached on the findings. An example of the analysis process, illustrating the development from meaning units to main category, is presented in Table 1.

**Table 1:** Example of the analysis process

Meaning unit	Code	Sub-category	Generic category	Main category
“What I’m thinking is that with language, you say one thing, but it gets completely misunderstood and that’s quite common nowadays, especially since many older people speak a different language.”	Language barriers	Beyond instructions: striving for clarity and trust	Addressing the patient’s needs	Professional discretion and coordinated support for sustainable decision making
“With experience and having worked for so many years, I feel very confident.”	Experience brings confidence	Learning the ‘Clinical Eye’: Intuition, experience and knowledge	The caring capacity of a PEN	Professional discretion and coordinated support for sustainable decision making
“The primary care is a classic example…we can never get through to them. We’ve tried calling for so long, or it’s just impossible to get an appointment.”	Lack of collaboration	Relying on the collaboration of other healthcare providers for safe decisions	Support, collaboration, and cooperation	Professional discretion and coordinated support for sustainable decision making

### Pre-understanding

The authors GBN, ES, CH, and AO have worked in prehospital emergency care, and all the authors have both experiential and contextual preunderstanding of caring for frail older patients in this setting. This first-hand experience has provided insights into the complexities of delivering emergency care outside hospital environments.

Throughout the analysis process, the authors continuously questioned and critically reflected upon their preunderstanding. This was achieved by actively identifying and discussing preconceptions about the phenomenon under study, as well as their assumptions regarding the challenges and nuances of providing care to frail older patients in prehospital settings. By maintaining a reflective approach, the authors strived to minimize bias and ensure that their interpretations remained grounded in the data rather than influenced by prior professional experience.

### Use of AI tools in manuscript preparation

Language editing and phrasing suggestions throughout the manuscript were supported via the AI tools ChatGPT (OpenAI, 2025) and Grammarly. All content was critically reviewed and approved by the authors, who take full responsibility for the final version.

## Results

In this study, PENs’ experiences of giving self-care advice and referring frail older patients to remain at home are described. The results are presented through one main category and followed by three generic categories. These generic categories consist of a total of nine subcategories (Table 2).

**Table 2:** Categories emerging from the data and present in the results

Main category	Generic categories	Subcategories	
Professional discretion and coordinated support for sustainable decision making	Understanding the patient’s needs	*Navigating uncertainty in patient ability*	
		*Beyond instructions: striving for clarity and trust*	
		*Respecting autonomy and self-determination while fearing the consequences*	
	The caring capacity of a PEN	*Learning the ‘Clinical Eye’: Intuition*,* experience and knowledge*	
		*Balancing dignity and safety through empathy*	
		*Negotiating power and responsibility*	
	Support, collaboration, and cooperation	*The fragile balance of relying on relatives*	
		*Balancing confidence and constraints within the internal organization*	
		*Relying on the collaboration of other healthcare providers for safe decisions*	

### Professional discretion and coordinated support for sustainable decision making

The main category illustrates PENs’ experiences of giving self-care advice and allowing frail older patients to remain at home when it is considered the best option. This requires empathy, a holistic perspective, and careful balancing of professional authority with patient and relatives’ autonomy. The findings also emphasize the importance of coordinated support, including structured assessment tools, interprofessional collaboration, and resources to manage uncertainty in decision-making.

#### Understanding the patient’s needs

Understanding the patient’s needs means that PENs need to be attentive and assess each patient’s cognitive, physical, functional, and communicative abilities, while also considering their autonomy and self-determination.

##### Navigating uncertainty in patient ability

PENs describe how patients’ cognitive, physical, and functional abilities are central to the decision-making process when giving self-care advice and allowing patients to remain at home. In this assessment process, these abilities act as signposts that help PENs navigate the uncertainty surrounding the final decision.

The cognitive ability of a frail older patient is perceived as particularly significant and emerges as an uncertain component in the assessment. PENs describe, for example, how signs of memory impairment or confusion can trigger an instinctive sense of caution. A patient who appears unable to process the information provided, forgets what has just been said, or lacks insight into their condition is perceived as more vulnerable and at risk. This evokes feelings of responsibility and concern that the patient, without a full understanding of the self-care advice. Such concerns often lead to the decision to transport the patient to the emergency department to ensure that they receive the necessary care.

I feel like I have been talking to a wall (…) you will not remember any of this. Leaving you here is not an option, as you will call again in three hours. (P1)

Frail older patients’ physically limited ability, characterized by multimorbidity, impaired balance, reduced mobility, and vision or hearing impairments, are perceived as a significant challenge to their ability to manage self-care advice and remain safe at home. PENs expressed uncertainty about whether frail older patients could follow self-care advice or seek help if their condition worsened, which created a sense of insecurity and an ethical as well as clinical dilemma that further complicated decision-making.

I usually feel confident in making decisions about prescribing or not prescribing, as well as providing self-care advice at home, just as I would for any other patient. However, if they have poor balance, are they even able to retrieve it from the cabinet themselves…? (P3)

PENs experience that frail older patients have significant limitations in their practical capacities. Patients are seen struggling to book appointments at primary care centers, navigate telephone queues, or use technical aids, further increasing the risk of isolation from appropriate healthcare services. This situation generates a sense of ambivalence among PENs, where a prominent concern is that patients who are not transported to the emergency department may refrain from seeking further medical care, even in the event of a deterioration in their condition.

##### Beyond instructions: striving for clarity and trust

The communication is perceived as a crucial factor in PEN decisions to provide self-care advice and leave patients at home. PENs describe uncertainty regarding whether their communication has been effectively received, particularly in cases involving language barriers. For them, clear and effective communication is a prerequisite for leaving a patient at home without compromising patient safety.

To feel secure in their decision, PENs need to perceive the patient as oriented, receptive, and capable of following the given instructions. When this is not the case due to language difficulties or other communication barriers, concerns arise about the risk of critical information being misunderstood or lost. The uncertainty regarding whether the patient can truly manage their situation at home thus becomes an inherent part of the assessment process.

PENs emphasize that communication is not only about delivering instructions but also about creating a dialogue in which the patient can confirm their understanding. A lack of feedback from the patient leaves PENs in a state of uncertainty and a sense of inadequacy. Strategies such as adjusting the pace of communication or writing down information are described as attempts to bridge this uncertainty.

Clear and confirming communication feels better. It’s not just us as healthcare professionals talking at them, but actually asking something like, ‘What did I just say?’ or ‘What are you going to do?’ and making sure they have understood. (P10)

Persistent uncertainty about whether information has been clearly understood highlights the complexity of communication with frail older patients receiving self-care advice. For PENs, communication is not only about giving instructions but also about fostering security and trust, as clarity can be crucial for the patient’s well-being. Effective communication builds trust and confidence, whereas failure reinforces uncertainty and heightens the moral responsibility for ongoing care.

##### Respecting autonomy and self-determination while fearing the consequences

PENs describe facing an emotional and professional balancing act when navigating the autonomy and self-determination of frail older patients. Older patients are perceived as expressing a strong desire to maintain their independence and integrity. At the same time, they may require guidance to understand that their current health condition might no longer allow for complete self-sufficiency. For PENs, this presents a weighty ethical dilemma. On the one hand, they seek to respect the patient’s wishes; on the other hand, they experience persistent concerns about what may happen if the patient does not receive the necessary care.

What I find the hardest is when someone refuses to go… and you just know that what I leave behind will not work. It won’t end well. (P3)

When a patient is fully oriented and aware of their decision, their will is described as taking precedence, even if it contradicts the PEN’s clinical judgment.

They are fully aware of what they are saying. Naturally, their will takes priority. (P8)

At the same time, PENs describe situations where some patients appear to recognize that they may not be able to manage at home yet still resist leaving the familiar comfort of their surroundings. This duality creates a difficult balance between honoring the patient’s wishes and addressing the need for medical care.

#### The caring capacity of a PEN

The caring capacity of PENs shapes both the provision of self-care advice and decisions about whether frail older patients can remain at home. This capacity is influenced by knowledge and experience, empathy, and the balance of power in the encounter, which together affect how advice is tailored and how confident PENs feel in their decisions.

##### Learning the ‘clinical eye’: intuition, experience and knowledge

PENs describe their confidence in providing self-care advice and safely allowing frail older patients to remain at home as something that develops over time and is grounded in accumulated clinical experience and professional knowledge. This process allows them to refine their “clinical eye” to identify which patients can manage at home. Experience is also seen to foster an intuitive “feeling” for which self-care advice is most effective, developed through years of practice.

…and then the experience builds over time, year after year, and you develop more of a sense of what works for the patients you meet. (P6)

Experience is linked not only to time spent in the profession but also to previous work backgrounds. Those who have worked in geriatric care report having a broader understanding of the needs of frail older patients, which increases their confidence in decision-making. Conversely, a lack of experience can create uncertainty, especially among newly qualified PENs, making collegial support crucial to ensure that no important aspects are overlooked.

Although PENs receive specialist training in prehospital emergency care, they feel insufficiently prepared to assess and care for frail older patients, and this lack of geriatric knowledge contributes to hesitation when providing self-care advice and leaving patients at home.

But we don’t really study much geriatrics, palliative care, or those kinds of courses. Of course, that would have been useful because the largest patient group we encounter in the AS is actually sick or frail older patients who are not acutely ill. (P4)

Furthermore, there is a perceived need for continuous professional development to remain up-to-date and confident in clinical assessments. PENs also express a desire for a deeper understanding of frail older patients’ long-term care needs, which they believe would enhance their ability to conduct more comprehensive assessments.

##### Balancing dignity and safety through empathy

PENs’ empathic approach toward frail older patients is characterized by a balance between ensuring the individual’s safety and preserving their dignity. In these encounters, PENs navigate a complex situation where both medical needs and the individual’s unique circumstances must be considered.

Creating a sense of security involves enabling patients to remain at home, as PENs experience that hospital transfers may pose risks such as cognitive decline, infections, and other complications. These risks must be weighed against the benefits of onsite treatment, requiring both medical competence and empathy for the patient’s wishes and abilities.

Helping them feel safe enough to stay at home… their best environment is their home, their safe space, and I want to find alternative and better solutions whenever possible. (P2)

Moreover, these situations present an ethical dilemma. Leaving a frail older patient at home may bring uncertainty about whether their health will deteriorate, while transporting them to the hospital often feels like an intrusion into their sense of security and dignity.

If you only consider the medical risks of going to the emergency department, they are enormous… older patients who go to the emergency department often die due to complications they acquire there. (P1)

PENs strive to create the best possible conditions for patients, even when it means thinking beyond conventional frameworks. This often blurs professional boundaries, as they find themselves advocating for patients in ways that extend beyond standard protocols. Allowing patients to remain at home when appropriate is described as an ethical and professional victory, though it can be both resource-intensive and emotionally demanding.

PENs describe striving to create the best possible conditions for patients, even when this means working beyond conventional frameworks and protocols. This can blur PENs boundaries, as they often advocate for and protect patients in ways not formally expected. The tension between following clinical guidelines and addressing individual needs creates a sense of navigating an ethical and professional gray area. Allowing frail older patients to remain at home is seen as an ethical and professional victory that preserves dignity and quality of life, though it is also emotionally demanding and resource-intensive.

It took time, and we had to make a few calls, but it was worth it… she was grateful for it in the end. The easiest choice would have been to put her in the ambulance and leave, but that just didn’t feel right. (P6)

##### Negotiating power and responsibility

PENs hold considerable power in encounters with frail older patients, as their clinical assessments may override patient wishes. PENs describe frustration in encounters with frail older patients and their relatives, particularly when expectations are perceived as unrealistic. The need to explain that a situation is not acute, despite the patient’s request for hospital care, often creates tension. This frustration is amplified when patients appear to understand the medical assessment but still refuse to accept it. When patient expectations cannot be met, a power imbalance arises between the patient’s disappointment and the PEN’s frustration. Frail older patients may hold a strong belief in the emergency department’s ability to immediately resolve all health concerns, only to be met with the recommendation to remain at home and take responsibility for their own care.

Then, of course, there is a sense of frustration… when you try to help them understand that their expectations simply cannot be fulfilled. (P2)

Providing self-care advice shifts responsibility onto patients, particularly when they refuse hospital transport. This transfer of power can leave PENs uneasy, not least because they feel obliged to document decisions thoroughly to protect themselves.

But in explaining it to them (frail older patients), you are also shifting responsibility onto them. So, I don’t know if I would call it uncertainty… rather, it’s about how sensitive this is. I (PEN) must make sure to record everything in the medical journal, in black and white, to prove that I have done everything I could. Otherwise, it might come back on me, and I could be held accountable for leaving them at home. (P3)

While denying transport may risk patients feeling disregarded and losing trust, PENs also highlight that providing self-care advice and leaving the patient at home can present an opportunity for reflection, allowing frail older patients to process and accept the given recommendations.

#### Support, collaboration, and cooperation

When deciding whether frail older patients can remain at home with self-care advice, relatives are crucial, both as a source of support that provides security for PENs and as a limiting factor when they are unable or unwilling to help, sometimes requesting hospital care instead. Furthermore, collaboration within the organization is important, through medical advice and decision support, as a resource for the PEN when assessing whether the patient can remain at home. In addition, cooperation with other healthcare providers, who can offer support such as follow-up care, can make it possible for frail older patients to stay in their homes. However, this requires coordinated care.

##### The fragile balance of relying on relatives

When frail older patients are assessed as suitable for remaining at home with self-care advice, relatives play a crucial role in creating a sense of security and ensuring that care plans are followed. For PENs to feel confident in their ability to leave frail older patients at home, the involvement and understanding of their relatives are essential. PENs stress that relatives must not only receive but also be able to act on self-care instructions. When relatives lack insight, have difficulty understanding the advice, or are themselves limited by age or illness, PENs feel greater uncertainty about leaving the patient at home.

As long as I know that we have a plan together and that they will follow that plan, then I feel fairly confident. (P4)

Relatives can serve as valuable resources in ensuring that self-care recommendations are adhered to. However, their role is not always without challenges, as emotional strain may affect their ability and willingness to provide adequate support. PENs describe cases in which relatives express feelings of exhaustion, which can hinder their ability to provide the expected level of care. In such cases, the decision to provide self-care advice is not solely a medical judgment but also an evaluation of social and relational factors.

Sometimes there is a social problem as well. It might be a strained relationship— ‘I can’t take care of my mother anymore. Just take her’… or ‘I am tired of my husband’ or ‘I can’t help him anymore’… so there is so much more to consider in these situations. (P9)

When providing self-care advice to frail older patients who lack relatives in their daily lives, PENs describe this as significantly influencing their decision-making. The absence of a support system creates a sense of inadequacy, as PENs are unable to provide the security and continuity that these patients need. As a result, it becomes difficult to justify leaving them at home with self-care advice. PENs must weigh the patient’s independence against the risk that a lack of support may compromise their health or safety.

##### Balancing confidence and constraints within the internal organization

One key factor in feeling secure when providing self-care advice and leaving frail older patients at home is the collaboration and support structure available within the AS organization. Access to collegial support, medical advice from physicians, and technological tools strengthens PENs’ confidence in their decision-making. PENs highlight the importance of having access to both collegial and medical support when uncertainty arises regarding self-care advice and leaving frail older patients at home. The ability to quickly consult physicians or colleagues within the organization is described as a crucial factor in fostering confidence in decision-making. Being able to discuss concerns and receive confirmation about their decisions provides a sense of security and reinforces PENs’ confidence in their assessments.

The times I have felt uncertain, I have wanted to check with someone, someone with higher medical authority, so to speak, to see if I am thinking correctly in this situation. (P3)

In addition to collegial and medical support, technological tools are also highlighted as a means to strengthen assessments and decision-making. One example is the use of triage tools that help determine the severity of a frail older patient’s condition. These tools are perceived as complementing clinical judgement and providing an objective basis for deciding whether to provide self-care advice and leave a patient at home.

We have West (a triage tool) that we can use as triage support to assess how potentially ill a patient is. (P5)

Despite access to support and decision-making aid, PENs emphasize that resource shortages remain a major challenge. They describe an internal conflict in which other patients may be in urgent need of care, while a lack of personnel creates additional strain. This results in a difficult balance between providing frail older patients with the support they require and managing limited resources efficiently.

We have limited resources. That means we don’t stay an extra 15 minutes just because they might need another phone call. (P3)

When PENs experience resource constraints, they are forced to prioritize, which creates feelings of frustration, stress, and ethical discomfort. They described it as emotionally burdensome to leave a patient with less support than they felt was needed, and worried about the potential consequences for patient safety.

##### Relying on the collaboration of other healthcare providers for safe decisions

For PENs to be confident in providing self-care advice and safely leave frail older patients at home, access to other healthcare providers and effective collaboration between different care institutions are of central importance. PENs highlight the community healthcare team as a critical support function in this collaboration. The ability of the community healthcare team to promptly follow up on patients at home creates a sense of security that benefits both patients and facilitates the decision to provide self-care advice. The availability of a community healthcare team, particularly during daytime hours, is perceived as reassuring, reducing the risk of unnecessary hospital visits and enabling patients to remain in their home environment.

It’s great that we have access to our community healthcare team, which can step in and follow up… If the community healthcare team didn’t exist, the situation would be much riskier. (P5)

Home care and home nursing services are also seen as important in decisions to leave frail older patients at home, but their resources are perceived as insufficient. PENs describe how limitations in available resources can lead to a shifting of responsibility between different care providers. The lack of adequate support creates a sense of inadequacy among PENs, who often have no alternative but to transport the patient to the emergency department.

Sometimes it feels like the municipal care services don’t want to take responsibility, and the emergency department doesn’t see it as an acute issue… and then these patients end up living in misery, and we don’t know what to do for them. (P4)

Despite the resources in place, PENs experience lack of coordination between healthcare providers, which complicates decision-making regarding self-care advice for frail older patients. Insufficient collaboration creates frustration and uncertainty among PENs. These shortcomings are seen as affecting not only their confidence in the healthcare system but also the quality of care provided to patients.

There seem to be gaps in the flow of information… something gets lost along the way, and we really need to collaborate better. (P7)

A well-functioning and integrated healthcare structure, connecting municipal care, primary care, and emergency care, is described as a key factor in creating confidence when deciding to provide self-care advice and allowing frail older patients to remain at home. Tools such as shared electronic health records and improved communication channels between different healthcare providers are highlighted as essential. This structure is perceived as enhancing collaboration between care institutions, ensuring that frail older patients’ care needs are met in a more effective and coordinated manner. Additionally, an integrated and well-coordinated healthcare system is seen as reducing the risk of information loss and misjudgement in patient care.

I would feel more confident with a shared medical records system and better collaboration between municipal and regional healthcare providers. (P9)

## Discussion

The present study explores PENs’ experiences in giving self-care advice and deciding to refer frail older patients to remain at home. The results show that by encountering frail older patients, PENs must carefully assess and respond to patients’ cognitive, physical, functional, and communicative abilities. Decision-making is shaped by clinical experience, empathy, power dynamics, and confidence in judgment while also requiring respect for patient autonomy. Relatives can act as key enablers or barriers in this process, depending on their ability and willingness to support the patient. Additionally, organizational collaboration, including access to medical advice, decision support, and cooperation with other care providers, is essential to ensure that patients can be safely left at home with appropriate self-care advice. Coordinated care structures are therefore crucial for strengthening patients’ capacity for self-management and enabling safe and sustainable care at home.

The result showed that clear and reciprocal communication between PENs, frail older patients, and their relatives is essential to ensure that self-care advice is understood and followed. At the same time, these interactions reveal a central dilemma: PENs must decide whether frail older patients can safely remain at home, balancing respect for autonomy with the responsibility to safeguard patient safety. Providing self-care advice without being certain that patients can or will, could create uncertainty and ethical strain for PENs. Backman et al. [15] highlighted that assessing frail older patients is particularly challenging, not only because of their multimorbidity and diffuse symptom presentation but also because of linguistic misunderstandings between healthcare providers and patients. Even with interpreters, PENs may remain uncertain about whether the frail older patient has truly understood, making the burden of leaving the patient at home even greater. Previous research has also shown that language barriers in healthcare generally increase the risk of patient safety incidents [16] and that older patients with cognitive impairment rarely express their needs clearly, which further complicates communication [17]. In such contexts, non-verbal communication, such as body language, eye contact, and tone of voice, becomes a crucial means of establishing trust and supporting patient understanding [18]. Taking time during consultations is also vital, as both frail older patients and relatives value attentiveness and confirmation that the information is fully understood [19]. Yet, the time-limited prehospital environment can make this difficult, adding to ethical stress and professional responsibility PENs feel when balancing the patient’s right to self-determination with the duty to prevent harm [20]. These findings suggest that communication skills are not only practical competencies but also integral to navigating the core ethical dilemmas in PENs’ decision-making about referring frail older patients to remain at home with self-care advice.

The findings also indicated that decision-making regarding the referral of frail older patients to remain at home with self-care advice is a complex process influenced by the PEN’s prior experience and knowledge. A lack of geriatric competence may lead to uncertainty and defensive decision-making, such as transporting patients to the emergency department out of fear of overlooking signs of clinical deterioration. This aligns with previous research highlighting the challenges of assessing frail older patients in the prehospital setting [21] and reflects the “defensive practice” often associated with moral distress, where clinicians choose the safer institutional option to mitigate personal accountability rather than fully honouring patient autonomy [22]. Conversely, another study showed that PENs felt more confident and competent after education, which strengthened their ethical reasoning and decision-making when advising patients with self-care advice [15]. This underscores not only the need for geriatric competence but also the importance of continuous ethics education, reflective exercises, and simulation-based training. Such initiatives can reduce moral distress, enhance resilience, and equip PENs with tools to manage the ethical dilemmas that arise in the complex balance between patient autonomy and safety [23]. High-fidelity simulations, particularly those involving ethically challenging scenarios, have also been shown to stimulate ethical reflection and strengthen PEN students’ preparedness for real-life decision-making [24]. Furthermore, core competencies developed during PENs education, such as clinical judgment, communication, and interprofessional collaboration, are essential for making safe and well-informed care decisions in prehospital emergency care [25], for example, concerning frail older patients. Moreover, the use of decision-support tools in the assessment of frail older patients can assist PENs in directing patients toward appropriate alternative care pathways rather than routinely transporting them to the emergency department. The application of such tools not only enhances clinical confidence but also enables PENs to apply their knowledge in a more structured and systematic manner. Structured decision-support systems have also been shown to improve the quality of care for older patients and contribute to more efficient use of emergency healthcare resources [21].

The findings showed that PENs must balance respect for frail older patients’ autonomy with their responsibility to ensure that self-care advice and decisions for patients to remain at home do not jeopardize health. Balancing respect for autonomy with the responsibility to protect frail older patients from harm can be understood through Sandman’s framework of prehospital ethics, where conflicts arise between what professionals *should* do, *must* do, and *can* do [26]. In situations where patient preferences are unclear or strongly shaped by relatives or other providers, PENs must weigh ethical ideals of autonomy (*should*) against their professional and legal obligations to ensure safety (*must*), while also working within the limits of organizational structures and available resources (*can*). This multi-layered conflict intensifies the ethical stress PENs experience, as any decision risks leaving one of these dimensions unmet [26]. From this perspective, ethical competence extends beyond individual decision-making to include the ability to recognize and navigate these structural tensions. Creating a patient-centered environment, where patients feel heard and respected, remains crucial [27]; however, PENs must also manage unrealistic expectations, such as the belief that the emergency department is always the only valid option [28]. These expectations amplify the conflict between the three dimensions of *should*, *must*, and *can*. In such contexts, communicative competence becomes not merely a clinical skill, but an ethical practice of negotiating competing demands with empathy and sensitivity to the relational and institutional setting [15].

The present study found that effective collaboration with community healthcare, home healthcare, and primary care is essential for keeping frail older patients safe at home. PENs emphasized that access to community teams supports decisions to leave patients with self-care advice, while poor coordination and unclear follow-up responsibilities caused frustration. This aligns with previous research, which demonstrated that healthcare professionals, despite having similar goals, often interpret patients’ needs differently due to organizational boundaries, thereby hindering effective collaboration [29]. Another study demonstrated that dialogue-based decision-making among PENs, primary care providers, and patients increases the sense of security in care decisions and enables older patients to remain at home with adequate support. Through open dialogue, experience and knowledge are shared, creating a more solid foundation for joint decision-making [30]. Such interprofessional collaboration is built on trust, shared responsibility, and a mutual commitment to tailoring care to the needs of the older person [30, 31]. In line with Sandman and Nordmark (2006) [26], these findings highlight that unclear organizational responsibilities create not only practical obstacles but also ethical challenges, as PENs may be left to carry the burden of risk without sufficient structural support. Strengthening collaboration is therefore not only a matter of efficiency but also an ethical necessity. Indeed, healthcare efficiency, meaning better use of resources to deliver safe and coordinated care, can be improved by up to 85% in ambulance services and 75% in home healthcare through meaningful knowledge exchange and cooperation [32]. Ongoing care relationships foster a shared understanding of patient needs, reducing the risk of misjudgement and unnecessary emergency department admissions. This underscores the importance of establishing collaborative structures that promote continuity, dialogue, and shared accountability among PENs, home healthcare staff, and primary care providers [31].

### Limitations

This study has some limitations. First, the relatively short duration of the interviews is a possible threat to credibility. No new perceptions emerged after ten interviews. Longer interviews might have increased the credibility of the study, but the number of different perceptions that emerged shows that the data were considered sufficient. Second, the study was conducted within a single ambulance service organization in southwestern Sweden, which may limit the transferability of the findings to other geographical regions or healthcare systems with different organizational structures and resources. Cultural and organizational differences in how prehospital care is delivered might impact the applicability of the results outside this context. Third, self-reporting bias is a potential limitation, as the interviews relied on participants’ recollections and perceptions. PENs may consciously or unconsciously emphasize certain aspects of their experiences or withheld negative experiences due to social desirability or fear of judgment.

Another limitation concerns the range of perspectives included. The study did not capture the views of patients or their relatives, whose experiences could have provided valuable insights and helped to triangulate the findings. Nor did it include other key stakeholders, such as general practitioners, municipal care staff, or emergency dispatch services, whose perspectives might have offered further depth in understanding the systemic factors influencing PENs’ decisions. Finally, future research should therefore explore the perspectives of frail older patients, their informal caregivers, and other stakeholders, and also examine how healthcare policies, organizational structures, and interprofessional collaboration frameworks influence PENs’ decision-making. Such studies could help identify both systemic barriers and enablers to safe, equitable, and sustainable prehospital care for frail older patients.

## Conclusion

This study highlights the multifaceted challenges PENs face when providing self-care advice and deciding to refer frail older patients to remain at home. Effective decision-making in these scenarios necessitates comprehensive assessments of patients’ cognitive, physical, functional, and communicative capacities. Clear and reciprocal communication among PENs, patients, and their relatives is essential to ensure that self-care advice is comprehended and adhered to. PENs’ clinical experience, empathetic engagement, and confidence in judgment are pivotal, especially when navigating the delicate ethical balance between respecting patient autonomy and ensuring safety. Organizational collaboration, including access to medical advice, decision support tools, and cooperation with other healthcare providers, is crucial to support safe, sustainable, and ethically sound decisions when advising frail older patients to remain at home with self-care advice.

## Supplementary Information

Below is the link to the electronic supplementary material.

Supplementary Material 1

Supplementary Material 2

## Data Availability

Data are available upon reasonable request. Interview data and analyses are in Swedish. The data are not publicly available due to privacy or ethical restrictions.

## References

[1] Statistics Sweden (SCB). After 60: a description of older people in Sweden. Demographic Reports 2022:2. https://www.scb.se. Accessed 4 June 2025.

[2] World Health Organization, Ageing and health. (2024). Available from: https://www.who.int/news-room/fact-sheets/detail/ageing-and-health. Accessed 4 June 2025.

[3] Hjalmarsson A, Holmberg M, Asp M, Östlund G, Nilsson KW, Kerstis B. Characteristic patterns of emergency ambulance assignments for older adults compared with adults requiring emergency care at home in sweden: a total population study. BMC Emerg Med. 2020. 10.1186/s12873-020-00387-y. 33267796 10.1186/s12873-020-00387-y PMC7709262

[4] Coats T, Conroy S, de Groot B, Heeren P, Lim S, Lucke J, et al. Prevalence of frailty in European emergency departments (FEED): an international flash mob study. Eur Geriatr Med. 2024. 10.1007/s41999-023-00926-3. 10.1007/s41999-023-00926-3 PMC10997678 38340282

[5] Ivic R, Kurland L, Vicente V, Castrén M, Bohm K. Serious conditions among patients with non-specific chief complaints in the pre-hospital setting: a retrospective cohort study. Scand J Trauma Resusc Emerg Med. 2020. 10.1186/s13049-020-00767-0. 32727586 10.1186/s13049-020-00767-0 PMC7391698

[6] Magnusson C, Hagiwara MA, Norberg-Boysen G, Kauppi W, Herlitz J, Axelsson C, et al. Suboptimal prehospital decision- making for referral to alternative levels of care – frequency, measurement, acceptance rate and room for improvement. BMC Emerg Med. 2022. 10.1186/s12873-022-00643-3. 35606694 10.1186/s12873-022-00643-3 PMC9125920

[7] Källberg AS, Berg LM, Skogli S, Bjurbo C, Muntlin Å, Ehrenberg A. Prevalence of frailty and associated factors in older adults seeking care at Swedish emergency departments. BMC Geriatr. 2023. 10.1186/s12877-023-04545-2. 38049748 10.1186/s12877-023-04545-2 PMC10694934

[8] De Brauwer I, Cornette P, D’Hoore W, Lorant V, Verschuren F, Thys F, et al. Factors to improve quality for older patients in the emergency department: a qualitative study of patient trajectory. BMC Health Serv Res. 2021. 10.1186/s12913-021-06960-w. 34521415 10.1186/s12913-021-06960-w PMC8442337

[9] Norberg Boysen G, Christensson L, Wireklint Sundström B, Nyström M, Jutengren G. Use of the medical emergency services by patients with suspected acte primary healthcare problems: developing av questionnaire measure patient trust in healthcare. Eur J Person Centered Healthc. 2016. 10.5750/ejpch.v4i.

[10] Höglund E, Schröder A, Möller M, Andersson-Hagiwara M, Ohlsson-Nevo E. The ambulance nurse experiences of non-conveying patients. J Clin Nurs. 2019. 10.1111/jocn.14626. 30016570 10.1111/jocn.14626 PMC8045551

[11] Krohn J-N, Singler K. Frailty and prehospital emergency care: implications for paramedic education. Age Ageing. 2025. 10.1093/ageing/afaf126. 40381318 10.1093/ageing/afaf126

[12] Elo S, Kyngäs H. The qualitative content analysis process. J Adv Nurs. 2008. 10.1111/j.1365-2648.2007.04569.x. 18352969 10.1111/j.1365-2648.2007.04569.x

[13] Tong A, Sainsbury P, Craig J. Consolidated criteria for reporting qualitative research (COREQ): a 32-item checklist for interviews and focus groups. Int J Qual Health Care. 2007. 10.1093/intqhc/mzm042. 17872937 10.1093/intqhc/mzm042

[14] Etikan I. Comparison of convenience sampling and purposive sampling. Am J Theoretical Appl Stat. 2016. 10.11648/j.ajtas.20160501.11.

[15] Backman T, Juuso P, Borg R, Engström Å. Ambulance nurses’ experiences of deciding a patient does not require ambulance care. Nurs Open. 2019. 10.1002/nop2.255. 31367400 10.1002/nop2.255 PMC6650689

[16] Schulson LB, Rodriguez JA, Cruz R, Flynn D, Fernandez A. Patient safety event risk and language barriers: a scoping review. Joint Comm J Qual Patient Saf. 2025. 10.1016/j.jcjq.2025.02.002 10.1016/j.jcjq.2025.02.002 PMC12124954 40089441

[17] Hill NL, Bratlee-Whitaker E, Jang H, Bhargava S, Sillner AY, Do J, et al. Patient-provider communication about cognition and the role of memory concerns: a descriptive study. BMC Geriatr. 2023. 10.1186/s12877-023-04053-3. 37259029 10.1186/s12877-023-04053-3 PMC10233998

[18] Wanko Keutchafo EL, Kerr J, Jarvis MA. Evidence of nonverbal communication between nurses and older adults: a scoping review. BMC Nurs. 2020;19(1):53. 32550824 10.1186/s12912-020-00443-9 PMC7298765

[19] Larsson G, Dagerhem A, Wihlborg J, Rantala A. Satisfaction among non-conveyed patients and significant others when discharged at the scene by the ambulance service: an exploratory cross-sectional survey. BMC Emerg Med. 2022;22(1):100. 35672702 10.1186/s12873-022-00659-9 PMC9171931

[20] Bruun H, Milling L, Mikkelsen S, Huniche L. Ethical challenges experienced by prehospital emergency personnel: a practice-based model of analysis. BMC Med Ethics. 2022. 10.1186/s12912-020-00443-9. 35962434 10.1186/s12910-022-00821-9 PMC9373324

[21] Vicente V, Svensson L, Wireklint Sundström B, Sjöstrand F, Castren M. Randomized controlled trial of a prehospital decision system by emergency medical services to ensure optimal treatment for older adults in Sweden. J Am Geriatr Soc. 2014. 10.1111/jgs.12888. 24916953 10.1111/jgs.12888

[22] Epstein EG, Hamric AB. Moral distress, moral residue, and the crescendo effect. J Clin Ethics. 2009;20(4):330–42. 20120853

[23] Rushton CH. Transforming moral suffering by cultivating moral resilience and ethical practice. Am J Crit Care. 2023. 10.4037/ajcc2023207. 37391375 10.4037/ajcc2023207

[24] Wihlborg J, Andersson U, Sterner A, Sandman L, Kängström A, Boysen GN. Stimulating ambulance specialist nurse students’ ethical reflections by high-fidelity simulation. Nurs Ethics. 2025. 10.1177/09697330241291162. 39403826 10.1177/09697330241291162 PMC12171055

[25] Wihlborg J, Edgren G, Johansson A, Sivberg B. The desired competence of the Swedish ambulance nurse according to the professionals - a Delphi study. Int Emerg Nurs. 2014. 10.1016/j.ienj.2013.10.004. 24210954 10.1016/j.ienj.2013.10.004

[26] Sandman L, Nordmark A. Ethical conflicts in prehospital emergency care. Nurs Ethics. 2006. 10.1177/0969733006069694. 17193801 10.1177/0969733006069694

[27] Rantala A, Forsberg A, Ekwall A. Person-centred climate and psychometrical exploration of person-centredness and among patients not conveyed by the ambulance care service. Scand J Caring Sci. 2018. 10.1111/scs.12516. 28892188 10.1111/scs.12516

[28] Booker MJ, Shaw ARG, Purdy S. Why do patients with ‘primary care sensitive’ problems access ambulance services? A systematic mapping review of the literature. BMJ Open. 2015. 10.1136/bmjopen-2015-007726 25991458 10.1136/bmjopen-2015-007726 PMC4442240

[29] Jones CD, Vu MB, O’Donnell CM, Anderson ME, Patel S, Wald HL, et al. A failure to communicate: a qualitative exploration of care coordination between hospitalists and primary care providers around patient hospitalizations. J Gen Intern Med. 2015. 10.1007/s11606-014-3056-x. 25316586 10.1007/s11606-014-3056-x PMC4370981

[30] Forsgärde ES, Svensson A, Rööst M, Fridlund B, Elmqvist C. The dialogue as decision support; lived experiences of extended collaboration when an ambulance is called. Int J Qual Stud Health Well-being. 2021. 10.1080/17482631.2021.1970095. 34427535 10.1080/17482631.2021.1970095 PMC8386744

[31] Forsgärde ES, Rööst M, Svensson A, Fridlund B, Elmqvist C. Support in acute situations when a community health nurse is called: experiences of older patients, their significant others, and involved healthcare professionals- a qualitative interview study. BMC Geriatr. 2023. 10.1186/s12877-023-04331-0. 37770856 10.1186/s12877-023-04331-0 PMC10537128

[32] Wells S, Tamir O, Gray J, Naidoo D, Bekhit M, Goldmann D. Are quality improvement collaboratives effective? A systematic review. BMJ Qual Saf. 2018. 10.1136/bmjqs-2017-006926. 29055899 10.1136/bmjqs-2017-006926

[33] Swedish Parliament. SFS 2003:460. Act concerning the ethical review of research involving humans. (2003). Accessed 4 June 2025. Available from: https://www.riksdagen.se/sv/dokument-lagar/dokument/svensk-forfattningssamling/lag-2003460-om-etikprovning-av-forskning-som_sfs-2003-460

[34] World Medical Association Declaration of Helsinki: ethical principles for medical research involving human subjects. JAMA. 2013. 10.1001/jama.2013.281053. 10.1001/jama.2013.281053 24141714

