# Supplementary material for: Balancing care needs - a qualitative study on prehospital emergency nurses’ experiences of providing self-care advice and home referrals for frail older patients
Source: BMC Emerg Med. 2025 Sep 22;25:183. doi: 10.1186/s12873-025-01355-0 (PMC12455764; doi:10.1186/s12873-025-01355-0)
Supplement: Supplementary file 1 — Supplementary Material 1 [file 12873_2025_1355_MOESM1_ESM.docx]

Supplementary file 1 – Interview guide

| \| **Type of questions** \|  \| \| --- \| --- \| \| **Opening question** \| *“Can you tell me about your experiences of giving self-care advice and referring a frail older patient to remain at home?”* \| \| **Follow-up questions** \| *“What emotions do you experience when you receive an assignment involving a non-urgent, frail older patient?”* *“How do you feel when providing self-care advice to a frail older patient?”* *“Do you feel that, in your role as a PEN, you have sufficient knowledge to assess the appropriate level of care and provide self-care advice?”* *“What challenges do you experience when giving self-care advice to frail older patients?”* *“What would you need to feel more confident when giving self-care advice to patients?”* \| \| **Probing questions** \| *“What do you mean by that?”* *“Could you elaborate on your answer?”* *“What feelings does that evoke?”* \| |
| --- | --- | --- | --- | --- | --- | --- | --- | --- |
